# Surgical experience for patients with serious mental illness: a qualitative study

**DOI:** 10.1186/s12888-021-03056-x

**Published:** 2021-01-20

**Authors:** Kate E. McBride, Michael J. Solomon, Tim Lambert, Sarah O’Shannassy, Catherine Yates, Jemima Isbester, Nick Glozier

**Affiliations:** 1 RPA Institute of Academic Surgery (IAS), Royal Prince Alfred Hospital and University of Sydney, PO Box M157, Sydney, New South Wales Australia; 2 Sydney Medical School, University of Sydney, Sydney, New South Wales Australia; 3 Surgical Outcomes Research Centre (SOuRCe), Sydney, New South Wales Australia; 4 ccCHiP, Charles Perkins Centre, University of Sydney, Sydney, New South Wales Australia; 5 Sydney Local Health District, Sydney, New South Wales Australia; 6 Brain and Mind Centre, University of Sydney, Sydney, New South Wales Australia

**Keywords:** Mental illness, Surgery, Qualitative, Patient experience, Surgical outcomes

## Abstract

**Background:**

People with serious mental illness (SMI) have significantly worse surgical outcomes compared to the general population. There are many contributing factors to this complex issue, however consideration of the surgical experience from the patient’s own perspective has never been undertaken. This lack of understanding prevents the provision of truly patient centred care and may limit the impact of potential improvement initiatives. Therefore this study aims to describe and better understand the surgical experience from the perspective of patients with SMI.

**Methods:**

Within this qualitative study, semi-structured, audio-recorded interviews were conducted between August 2019 – June 2020, with 10 consenting participants with SMI who had surgery in the previous 2 years. A thematic analysis approach was used to explore both the positive and negative aspects of the participant’s surgical experience commencing from pre-operative consultation to hospital discharge and follow-up.

**Results:**

Four main themes and related subthemes emerged including i) the perceived lack of mental ill health recognition, ii) highly variable patient and clinician interactions, iii) the impact of healthcare services, and iv) strategies for improvement.

**Conclusion:**

Surgical patients with SMI want to be treated like everyone else whilst still having their mental ill health acknowledged and proactively managed despite this rarely occurring, which is valuable information for all surgical teams to consider and learn from. Participants were able to describe several readily implementable strategies to potentially improve their care and overall surgical experience, and as such highlight considerable opportunities for these to be tested and evaluated for this underserved patient group.

## Introduction

Serious mental illness (SMI) affects 3 % of the population and includes disabling forms of depression and anxiety, as well as psychotic disorders such as bipolar and schizophrenia [1]. Like everyone, people with SMI may require surgery during their lifetime. Unfortunately however, they have significantly worse surgical outcomes including greater post-operative morbidity, longer stays in hospitals, and more re-admissions compared to the general population [2–4].

There are many contributing factors to this highly complex issue. From a health system perspective, many health professionals including surgeons still demonstrate stigmatising behaviours towards patients with SMI [5–7]. Indeed surgeons are purportedly less confident in looking after their patients mental health needs [8], less frequently enquiring about their patients mental health [8, 9], and more likely to overlook comorbid psychiatric disorders [9, 10]. Psychiatric co-morbidity is also managed differently pre-operatively compared to other medical co-morbidities [8, 10, 11]. From a patient level perspective, people with SMI have a higher burden of chronic disease [12, 13] and co-morbidities [14, 15] that may contribute to their poorer surgical outcomes. They also face social and occupational challenges that can make negotiating a complex, and at times expensive, health system arduous, which may exacerbate their mental illness symptoms [12–16]. What is unknown at the patient level, is how the surgical experience of these patients from their own perspective might influence surgical outcomes and their SMI. Whilst such experience of care has been investigated within primary and general tertiary medical care settings, and incorporates themes of access difficulties, communication challenges, exclusion from decision making and a need for holistic care [17, 18], no evidence can be found within the surgical context specifically. This lack of understanding is detrimental to providing surgical services that are truly patient centred and responsive to their needs.

As such the primary aim of this qualitative study is to describe and better understand the surgical experience from the perspective of those patients with SMI. The secondary aim is to utilise the information provided to guide the development of interventions aimed at improving the surgical experience for this vulnerable patient group.

## Methods

Consultation on the qualitative study design was undertaken at a consumer forum, the Sydney Local Health District (SLHD) Lived Experiences Advisory Panel (LEAP) with members generously providing valuable feedback. Ethics approval to conduct this study was granted by the Royal Prince Alfred Ethics Review Committee (X19–0140). Written or recorded verbal consent was provided by all participants.

### Setting and participants

Commencing in July 2019, participants were recruited via an information flyer, which was distributed at the LEAP consumer meetings and displayed at Community Health Centres and Health Facilities (both psychiatric and non-psychiatric) treating patients with SMI within the SLHD in Sydney, Australia. Inclusion criteria were people: 1) with self-reported serious mental illness who were or had previously been seen by a psychiatrist or attending secondary mental health care services, and 2) who had undergone surgery (including an anaesthetic and an invasive procedure) within the last 2 years. Exclusion criteria were people who were unable to communicate in conversational English or those experiencing an acute episode of mental or medical illness.

### Interviews

Following consent, interviews were conducted at the RPA Institute of Academic Surgery, Sydney, Australia or over the telephone. Participants were interviewed by two study investigators (KM + SO or CY), and recorded using a digital recorder and/or with manual notes taken. The interviews were semi-structured and explored both the positive and negative aspects of the participant’s surgical experience and “journey” commencing from pre-operative consultation to hospital discharge and follow-up. The interview schedule is outlined in Table 1 and was initially derived from several sources [17, 18], and then refined by the authors with expertise in both psychiatry and surgery, following consultation with colleagues and a consensus meeting. Participants received a $50 supermarket voucher at the completion of their interview in recognition of their contribution. Participants were also asked basic demographic information to ensure purposive sampling included a range of ages, genders, and surgical admission and procedure types. To mitigate the potential risk of harm to participants, a study Distress Protocol was developed and implemented. Participants were given the opportunity to review and edit a transcribed copy of their interview prior to data analysis.

**Table 1:** Interview schedule

**PRE-OPERATIVE SETTING**	
1. Why did you need to have surgery? How did you choose your surgeon? 2. What was it like talking with the surgeon? What was your impression of them? 3. How did the surgeon explain to you that you needed to have surgery? 4. How did the surgeon approach your mental health during this discussion? How did you feel about this? 5. What was the preparation and planning like for your surgery and going to hospital? Who did you speak with? 6. How did you feel about having surgery in relation to your mental health?	
**POST-OPERATIVE SETTING**	
7. How do you feel now about having your surgery and the time you were in hospital? 8. How was your mental health managed when you were in hospital for your surgery? 9. How did you feel talking with the ward staff when you were in hospital? 10. How did you feel about going home after your surgery? 11. How did your admission to hospital for surgery compare with other hospital admissions you have had before? 12. If there was one thing you could change about your experience with having surgery, what would it be? 13. Is there anything else you would like to add that we haven’t covered today?	

### Data analysis

Thematic analysis as described by Braun and Clarke was used in this study [19]. This flexible method was selected for providing a rich thematic description of the entire data set, which was identified as being critical given this is an under-researched area and the views of the participants were not known. The overall theoretical approach taken was that of a realist position, where a straightforward relationship between the experience and meaning was theorized [20], with semantic themes being developed from an inductive approach. This involved reading and re-reading the transcripts to define and refine the codes and themes with the interviews continuing to be performed until data saturation was reached. This occurred at participant #8 with two subsequent interviews conducted to ensure no new information could be obtained. Specific responses were manually coded using Nvivo (version 10; QSR International, Doncaster, Victoria, Australia) and independently assigned by two investigators (KM and CY). Equal weight was given to all data to develop as many codes as possible. Once the codes were determined, the investigators worked together to sort the codes into main themes with several corresponding sub-themes developed when the data supported it.

## Results

Ten participants (five women and five men) consented to participate with their demographic characteristics outlined in Table 2. The mean age was 47 years (28–70). The participants described having a range of mental illnesses including schizophrenia, schizo-affective disorder, bipolar disorder, complex post-traumatic stress disorder, obsessive compulsive personality disorder, severe anxiety, and severe depression with some participants reporting having more than one diagnoses. Six participants had elective and four had emergency surgery within various surgical specialties and admission types.

**Table 2:** Demographic characteristics of the participants

Characteristics	***n***
*Sex*	
Female	5
Male	5
*Age*	
18–39	4
40–64	4
64 years +	2
*Surgical Admission*	
Elective	6
Emergency	4
Day-stay	4
Overnight	6
*Surgical Specialty*^a^	
Colorectal / General Surgery	6
Orthopaedic	3
Upper GI	2
Oral Surgery	1
Gynaecology	1
*Serious Mental Illness*^a,b^	
Schizophrenia	3
Bipolar Disorder	3
Schizoaffective Disorder	2
Post-traumatic Stress Disorder including complex	2
Severe Depression	2
Psychosis	1
Obsessive Compulsive Personality Disorder	1
Severe Anxiety	1

^a^Participants may have reported more than one category

^b^As described by participants. Note one participant declined to provide this information

Four main themes and related sub-themes were extracted from the data to describe the participants’ experience of having surgery including: i) the perceived lack of mental ill health recognition, ii) highly variable patient and clinician interactions, iii) the impact of healthcare services, and iv) strategies for improvement. Illustrative quotes from participants are included in the themes below and further summarised in Table 3**.**

**Table 3:** Illustrative participant quotations by theme

**Theme 1: Perceived lack of mental ill health recognition**	
*[P1] “The only thing that came up about mental health was because of the bowel obstruction, … ..and they thought the medication I’m on, the antipsychotics, could be a potential cause, … .so they brought that up. But other than that he didn’t talk about me having a mental illness at any point. He never asked or questioned it”.* *[P7] – “I didn’t talk about it. See I think not to, well you’re damned if you do and damned if you don’t. And no, he didn’t ask, no.”* *[P5] “I mean, how would they even know that I’m going through something already? So it becomes two struggles. One is the internal mental health struggle, and the external surgery struggle. I think when people are more attentive, more thoughtful and more caring. I felt if somebody is crying, definitely the person needs somebody to have a word with them, or at least ask them are you all right? That would make a difference to me”.* *[P2] – “Like, if the doctor said, you know, “If you make progress in the next two weeks, then the week after we’ll decide that you can go home”. Rather than, kind of, leaving it in the air, sort of thing … just indicators that might give you an idea of the timeline”.*	
**Theme 2: Highly variable patient and clinician interactions**	
*[P8] - “I really was very pleased that they brought it up and that they gave it such credence in their practice and diagnosis, and has always asked me how it’s going with my psychiatrist and how things are … .. yeah, but it’s not widely acknowledged just as yet, I think, so I was really pleased that they had that attitude, so it helped me as well”.* *[P5] – “Fortunately my surgeon was a very jolly person. He was always joking around and because I felt so comfortable with him, I was also joking and so I think that made a lot of difference because he took everything so lightly and he was very talkative and friendly. It calmed my anxiety and took my mind away from my mental health”.* *[P7] – “I trusted them, they’re the professionals. I’ve got no role here. They’re the professionals. I think it broke my trust in the system, my trust in the hospital system. I feel like I’m powerless in that situation”.* *[P3] – “No I just told my friends. I just kept everything to myself … .nobody really asked about how I’m feeling …*. I *always had people to talk to but I don’t think anybody came up from the mental health side of it and spoke to me how I’m feeling and how am I coping and stuff like that”.* *[P5] – “I tell you, the opinions of the people, the judgmental attitude of the people, that creates a lot of stress. I want to tell them about my mental health problems, but there’s nobody to listen to me”.* *[P4] – “I remember crying the day before surgery, which was a sign I wasn’t well as I don’t normally cry. I was also crying afterwards, which wasn’t a normal reaction for me. … with retrospect the crying was a big red flag for me but no one asked me about it. I didn’t see anyone like a social worker or psychologist.”*	
**Theme 3: Impact of healthcare services**	
*[P7] – “As I said I felt like I was just a number – just in and out sort of thing”.* *[P5] – “I think I was the only one crying, because I think it gets too much when you have a mental illness, plus you have this horrible pain and nobody is taking you in because everybody in ED is in their particular emergency”.* *[P6] – “It doesn’t take much to be able to read a man’s medical history … .and determine that. I believe, they ought to go one further in investing in keyword search. And these keywords, this man suffers from a primary health diagnosis, mental health … they should be highlighted on the screen …*.” *.* *[P2] – “The system. It does feel at times that I have to advocate for myself. Maybe that’s a good thing. Maybe its like a double-edge sword.”*	
**Theme 4: Strategies for improvement**	
*[P6] – “They’re all referring to the same database, but its perhaps not adapted to a person who is upset, and is going to respond differently to the next person in the queue, behind me. This issue, that’s what I’d like to see changed”.* *[P4] – “It would have been good for a simple message about if your mood changes just to watch out for it. There was nothing about mental health. I wasn’t given any numbers to call or what to do if I had any drastic changes in my mood.”* *[P8] –* ***“****And if they know that there’s someone they could ring in the hospital or after they’ve visited they might even do that, rather than do it right at the time. It’s a sort of a process for everyone really, it can be different for everyone.”* *[P4] –* ***“****It would help if someone helped coordinate what is going to happen, especially when you go home”.* *[P8] – “Because, I mean, I’m very aware that people can present like nothing’s going on and that things are going really badly inside, so I think always bringing it up and giving people the option, even if they don’t say there’s anything wrong at that time, if you give them the option to contact someone if things do start to go wrong, that would be a good thing. I think it’s always good to have a safety net there”.*	

### Perceived lack of mental ill health recognition

On the whole, participants described a lack of consideration of their mental health comorbidity during the provision of surgical care, reflected in two sub-themes: *i) focus on physical health* and *ii) focus on in-hospital processes.*

#### Focus on physical health

Participants reported their mental illness was rarely identified, acknowledged or considered throughout all stages of their surgical admission. During the pre-operative consultation almost all participants advised their surgeon did not ask about their mental health condition. This included partipants who experienced an emergency surgical admission directly caused by a psychotic episode and participant’s on medication known to interact poorly with commonly used anaesthetic drugs.

The provision of care and information was primarily focused on their physical wellbeing, existing chronic physical comorbidities, wound management, pain relief or rehabilitation, with little or no attention given to addressing the patients’ mental ill health, even for those who described being visibly distressed.

[*P4] “The staff were just all focused on the medical or clinical side of things rather than the mental health side of things. There wasn’t anything about mental health. I wasn’t given any numbers to call or what to do if I had any drastic changes in my mood.”*

Some acknowledged this was compounded by the participants themselves being focused on their physical symptoms, and subsequent need for surgical treatment, which superseded any consideration being placed on their mental illness in relation to their surgery. Many participants also advised they weren’t aware their surgical treatment could impact on their mental health, reflecting the lack of planning and preparation they were given.

*[P3] “I was just concerned about my foot to be honest with you. That was the last thing I was worried about … . I don’t think so because I was in so much pain with my foot.”*

#### Focus on in-hospital processes

Participants also described their care being entirely focused on their in-hospital admission. Little to no discussions were recalled being had about how their surgical treatment might impact on their mental health over time, or what to do if their mental health should decline following discharge from hospital. Accordingly, there was no specific mental health information provided to participants on discharge, and inconsistent connections were made with the participant’s community mental health team. Overall this communication was described as being left to the participants to manage for themselves.

*[P4] – “All my mental health issues started once I left hospital. I was mostly fine in hospital because it was very busy and there was a lot going on … If I had been referred to mental health support after I left hospital, it may have helped. It might have prevented my admission to the psych hospital.”*

### Highly variable patient and clinician interactions

Participants described experiencing varying levels of engagement and support from a range of clinicians involved in their surgical care, which is incorporated into two opposing subthemes: i) clinicians enhancing the surgical experience and ii) clinicians diminishing the surgical experience.

#### Clinicians enhancing the surgical experience

It was important to participants they were treated like everyone else by clinicians and this was described as being the common experience. The capacity of clinicians to positively impact on the participants was considerable. Although encountered rarely, there were examples recounted where clinicians were able to make participants feel comfortable to talk about their mental illness by just acknowledging it without fanfare or by sharing their own experiences. Similarly, the use of humour as a tool to make participants feel comfortable and to alleviate their distress or anxiety, or to minimise the symptoms of their mental illness was highlighted as enhancing their experience. Finally participants valued it when clinicians conveyed compassion and empathy towards them, and acknowledged the challenges associated with their mental illness including the links with their physical health.

*[P6] – “He came round to everyone one of us, and I heard him speak the same to me, as he did to the patient next to me, and the one a bed over. He cracked a joke, which lightened the humour … ..and I thought, gee, you know your job well. Gee, you handled that well. You lightened the mood, got us all cackling.”*

*[P1] – “Actually only to one nurse, because she was cool. She goes, I used to be a psych nurse and then I told her about myself and stuff like that, but the whole nursing staff knew because they gave me my medications so they knew anyway, so I didn’t have to talk about it. … .I just wanted to be treated normally. And they did, they treated me the same”.*

#### Clinicians diminishing the surgical experience

Conversely, participants described their interactions with a range of clinicians negatively impacting on their mental health. Detrimental communication styles and behaviours demonstratedby some clinicians included being judgemental, dismissive, impatient or unapproachable. This resulted in participants feeling unable or uncomfortable to talk about their mental illness and further isolated during their surgical admission.

*[P2] – “He [the surgeon] couldn’t relate to me. He didn’t quite get my lifestyle. So it was a bit awkward there. And then, this is after the surgery, he had a team of young men and they just came in, kind of, wham bam thank you ma’am, you know, they spent 30 seconds looking at me, and asked, like one question. And they were super satisfied with that, like going “Yeah. That’s cool.” And it just seemed like they were full of bravado. And that they were on the way to the pub to just be like boys really”*

*[P2] – “To my knowledge the time I saw the surgeon was just before the surgery. Its interesting, because of all the different types of roles of people in the hospital, the surgeons gave me the worst impression. I didn’t feel comfortable asking questions. Because the manner is just get in there, and get it done as fast as possible … I don’t think there was opportunity for that”.*

### The impact of healthcare services

Participants described their experiences in relation to the healthcare system itself, which they felt either exacerbated symptoms of their mental illness or did not accommodate their unique needs, or conversely had a positive and beneficial impact on their wellbeing. This included two sub-themes: i) hospital environment and ii) management of patient information.

#### Hospital environment

The hospital environment was identified as contributing significantly to the participants’ surgical experience. From a negative aspect, this involved the very busy, highly systematised nature of surgery giving the participants the impression there was either no time or that it wasn’t appropriate to raise the topic of their mental illness. This was particularly the case for those patients who underwent day surgery or were admitted through the emergency department. Similarly, participants described the delivery of surgical care as being overwhelming and causing a level of sensory overload at times, which was triggering to symptoms of their mental illness.

*[P5] – “The infrastructure is important with mental health issues, external noise is unbearable. I mean, I can’t take a lot of noise … ..When you are a mental health patient the environment can stimulate, trigger a lot of things. So in fact I think it should be different, not so different, I mean it should still be inclusive, but at the same time more thoughtful about the environment, like quiet and peaceful instead of somebody banging the door all the time because it triggers.”*

Conversely, participants also described the positive impact the hospital environment had on their mental health. This encompassed their shared hospital accommodation on the wards not only contributing to them feeling included and like everyone else, but also offering them an opportunity to bond and share their experiences with other patients. The participants overwhelming described their experience of the surgical environment to be more caring and liberating compared with their experiences in solely psychiatric environments.

*[P2] – “I’m in the room with three other guys, and like, that was great fun talking with the other guys … .I was able to relate to the other patients. It requires a bit of honesty. And there’s some deep feeling amongst the patients … .And you get this, kind of, deep feeling that it’s like life and death here. So, yeah, that made it a bit easier to talk about a serious matter like mental illness. I became mates with a lot of them”.*

#### Management of patient information

Participants described management of their personal information as being a considerable and ongoing source of frustration. As a result of their mental illness, participants advised having multiple hospital admissions over many years which required them to constantly repeat their medical history and personal information. The lack of integration of information systems between hospitals, and between psychiatric and non-psychiatric health services was highlighted as being unnecessary, distressing and a process which is also exacerbating of their mental illness symptoms.

*[P6] – “If you’re dealing with a person with a diagnosed mental illness, it’s not the same as the Fred, who followed me in the queue, who is not subject to those particular symptoms; and was quite amenable to being asked pedantic, inane, simple, basic questions, that he knew you had the answer to already on your blue monitor.”*

*[P2] – “But I’ve just gotten so used to just giving my history again, and again, and again, and again.”*

### Strategies for improvement

Participants were able to articulate a number of strategies they felt would potentially improve the surgical experience for patients with SMI, which fittingly intersect with the other themes identified in this study and include i) mental health screening, ii) a mental health flag within the electronic medical record (eMR), and iii) the availability of a patient advocate.

#### Mental health screening

Participants suggested routine mental health screening should take place across different stages of their surgical treatment. This included pre-operatively, whilst in hospital and on discharge with varying methods proposed. It was evident for the small number of participants who encountered proactive consideration of their mental illness, their overall experience was greatly enhanced by it. This approach was highlighted as one that could potentially improve all of the challenges identified by participants in this study. In particular, it would not only support clinicians to better recognise and proactively manage mental illness but it would also enhance interactions with patients and support them to feel less alienated in the surgical environment.

*[P1] - “It should be just like saying you have high blood pressure so you should see this person, yeah it should be the same. So you’ve got schizophrenia, take an interest and go, okay, how are you feeling or maybe have a post-surgery sit down with a surgeon who is trained in mental health as well”.*

*[P4] - “Maybe having the standard anxiety and depression questionnaire to be filled out on admission and discharge for every patient to see if there are any changes or obvious concerns. This might just flag when something isn’t quite right with a person.”*

*[P8] – “I think what helped me was having really good preparation and having informed and supportive professional people, like my surgeon and my psychiatrist … . because it’s a time of very big mixed emotions after surgery and that can trigger all sorts of things.”*

#### Mental health flag within the eMR

Participants suggested having a flag implemented within the eMR to indicate a patient has a mental illness. Although potentially in tension with their desire to be treated like everyone else, this would serve to overcome their ongoing frustration of having to repeat their medical history and personal information for every hospital admission. It would also highlight their illness to clinical staff, which again might facilitate more proactive conversations and the delivery of care which is more responsive to their needs.

*[P6] – “A keyword search should be automatic for a person with a diagnosed mental illness, and it should pop up … ..be polite, and respectful, and kind; … ..don’t ask him the repetitive questions that you know the answer to if you read the screen in front of you”.*

#### Dedicated patient advocate

Having the support of family and friends was described by participants as being extremely important to their mental health, however many participants advised they did not have this assistance. They felt particularly vulnerable and isolated, which contributed to them being unable to talk to anyone about how they were feeling mentally or to clarify or raise questions about their surgical care. Participants suggested that having a dedicated advocate could assist future patients in overcoming many of the challenges they experienced. This included navigating the health system, initiating discussions about their mental illness, raising questions or concerns about their surgical care, providing linkages for patients once they had been discharged from hospital and critically, to check on their mental health status throughout their surgical journey.

*[P7] - “Well, I’d have an advocate, and I’d make sure that – I mean they can’t come in the operating room with you – but they can get feedback from you when you come out and they might be able to act on it … ..I don’t think I’d come to hospital again unless I had an advocate.”*

## Discussion

This qualitative study provides a detailed description of the surgical experience for patients with SMI. Four main themes were identified in the data including the: i) perceived lack of mental ill health recognition, ii) highly variable patient and clinician interactions, iii) the impact of healthcare services, and iv) strategies for improvement.

There was no indication in this study that participants experienced self-evident prejudice or discrimination during provision of their surgical care. Being treated like everyone else was important to them, and they were. However, with stigma being defined as incorporating problems of knowledge (ignorance), attitudes (prejudice), and behaviour (discrimination) [21], it is apparent challenges relating to the former still persist within surgical practice. Participants reported their prevailing experience to be one where their mental illness was not acknowledged, recognised or prioritised, with only a few surgeons described as being knowledgeable in this area. This supports existing evidence suggesting that surgeons, and clinicians more broadly, still disengage from patients with mental illness [7–11, 22] and unfortunately reinforces the known alienation and social isolation strongly associated with SMI [12].

Despite this, the participants in this study clearly described a desire to have their mental ill health acknowledged during their surgical care. Initiating this themselves is likely made challenging by known difficulties demonstrated in other clinical settings including a lack of health literacy, problems in communicating their health concerns and experiences of information overload [17, 18, 23, 24]. In the surgical setting, it may also be due to their immediate focus being on their surgical treatment rather than any emerging or potential mental health challenges. It is critical this preference is highlighted and made known to surgeons, and indeed all clinicians treating these patients, given they have been shown to incorrectly assume they either can’t ask patients about their mental health or that it would be inappropriate [8]. The findings of this study overwhelming demonstrate this not to be the case, and underscores the dissonant tension between patients wanting to be treated like everyone else but to also have their mental illness acknowledged, which is a consideration that must be navigated carefully. Furthermore, the range of both positive and negative encounters described by the participants clearly depicts the essential role clinician’s play in influencing the overall surgical experience and mental health status for patients, which is in keeping with evidence surrounding the importance of consumer-provider relationships and the breaking down of the conventional power demarcation toward one of mutual respect [23]. The opposing tension observed within the role clinicians play is one that certainly warrants further investigation to better understand the driving factors and context in which they emerge.

The challenges described by participants surrounding the surgical environment and management of patient information is in keeping with known literacy deficits for patients with SMI [24] along with the exacerbation of their SMI symptoms. The impact of this on the capacity of patients to not only give informed consent [25], but to navigate the highly systematised nature of surgical care in general should not be overlooked or underestimated.

Finally, the suggestion by participants to have dedicated advocates to assist patients with their surgical journey is in keeping with evidence that has demonstrated the success of this model in other medical settings, particularly using peers as the advocates [26–28], and is something that warrants further investigation in the surgical setting. Accordingly, there is also emerging evidence on the effectiveness of transitional interventions for patients being discharged from hospital that might also be effective in the surgical setting [29].

This study has some limitations. Firstly, the findings should be considered within the context of the participants, which included a small sample size, and the researchers included in the study. As the participants volunteered for the study or were referred by clinicians, and were a heterogeneous group in terms of their mental health diagnoses, it may be possible the cohort interviewed were biased or had particular experiences that compelled them to participate. As such the experiences they described may differ from the experiences of other individuals with mental illness, and the findings do not allow conclusions to be generated for specific mental health diagnoses. Similarly, only participants with conversational English were interviewed, which may have limited the information gathered. Finally although none of the research team were involved in or aware of the participants’ surgical treatment, their own experiences and expertise within either surgery or psychiatry is likely to have influenced their interpretation of the data.

There are a number of implications of the study findings. First and foremost it has highlighted there is considerable opportunity to enhance the surgical experience for patients with SMI. Organisational change is needed to proactively approach and better integrate the management of a person’s mental health status before, during, and after their surgical treatment. Certainly the experience of undergoing surgery is a major life event for anyone, and one that is known to have a general risk for further medical or psychiatric admissions being required with the window for this potential inevitability being many months [30]. Knowing this, it is unacceptable that mental health monitoring and follow-up is not implicit in surgical discharge planning. Furthermore, many clinicians involved in the surgical care of patients will benefit from this information and will hopefully provide an impetus for self-reflection regarding their interactions and provision of information and care to this patient group. It also highlights there is a need for further education and training to be implemented within this area. Indeed the participants in this study were able to identify and articulate several highly feasible strategies for enhancing their care, which should be explored in detail, implemented and evaluated where possible. More evidence is needed in this area with a paucity of studies focusing on testing interventions to improve surgical outcomes for patients with SMI. Accordingly, this study demonstrates that consumers have a valuable role to play in providing key information not only regarding their experiences, but also in developing strategies for how it could be improved and they should be further involved in this process.

In conclusion, this study provides unique insights into the surgical experience for patients with SMI including their desire to be treated like everyone else whilst still having their mental ill health acknowledged and proactively managed as part of their surgical care, which is currently not the pervasive experience. It also highlights important information that is valuable for all clinicians involved in their care to consider, and learn from. Most critically, the identified strategies should be used to guide the development, implementation and evaluation of interventions aimed at improving the management of comorbid SMI and the overall surgical experience for this underserved patient group.

## Data Availability

The datasets used and/or analysed during the current study are available from the corresponding author on reasonable request.
